# Inhibition of Filamentous Thermosensitive Mutant-Z Protein in *Bacillus subtilis* by Cyanobacterial Bioactive Compounds

**DOI:** 10.3390/molecules27061907

**Published:** 2022-03-15

**Authors:** Manisha Gurnani, Prangya Rath, Abhishek Chauhan, Anuj Ranjan, Arabinda Ghosh, Rup Lal, Nobendu Mukerjee, Nada H. Aljarba, Saad Alkahtani, Vishnu D. Rajput, Svetlana Sushkova, Evgenya V. Prazdnova, Tatiana Minkina, Tanu Jindal

**Affiliations:** 1 Amity Institute of Environmental Sciences, Amity University, Noida 201301, India; manishagurnani10@gmail.com (M.G.); prangyarath71@gmail.com (P.R.); 2 Amity Institute of Environment Toxicology and Safety Management, Amity University, Noida 201303, India; tjindal@amity.edu; 3 Academy of Biology and Biotechnology, Southern Federal University, 344006 Rostov-on-Don, Russia; rvishnu@sfedu.ru (V.D.R.); terra_rossa@mail.ru (S.S.); prazdnova@sfedu.ru (E.V.P.); tminkina@mail.ru (T.M.); 4 Department of Botany, Microbiology Division, Guwahati University, Guwahati 781014, India; dra.ghosh@gauhati.ac.in; 5 Department of Zoology, University of Delhi, New Delhi 110007, India; ruplal@gmail.com; 6 Department of Microbiology, Ramakrishna Mission Vivekananda Centenary College, Kolkata 700118, India; nabendu21@rkmvccrahara.org; 7 Department of Health Sciences, Novel Global Community Educational Foundation, Hebersham, NSW 2770, Australia; 8 Department of Biology, College of Science, Princess Nourah bint Abdulrahman University, Riyadh 11671, Saudi Arabia; nhaljarba@pnu.edu.sa; 9 Department of Zoology, College of Science, King Saud University, Riyadh 11451, Saudi Arabia

**Keywords:** alpha-dimorphecolic acid, docking, Z-ring, antibacterial drugs, agar well diffusion assay, molecular dynamics, simulation

## Abstract

Antibiotic resistance is one of the major growing concerns for public health. Conventional antibiotics act on a few predefined targets and, with time, several bacteria have developed resistance against a large number of antibiotics. The WHO has suggested that antibiotic resistance is at a crisis stage and identification of new antibiotics and targets could be the only approach to bridge the gap. Filamentous Temperature Sensitive-Mutant Z (Fts-Z) is one of the promising and less explored antibiotic targets. It is a highly conserved protein and plays a key role in bacterial cell division by introducing a cytokinetic Z-ring formation. In the present article, the potential of over 165 cyanobacterial compounds with reported antibiotic activity against the catalytic core domain in the Fts-Z protein of the *Bacillus subtilis* was studied. The identified cyanobacterial compounds were screened using the GLIDE module of Maestro v-2019-2 followed by 100-ns molecular dynamics (MD) simulation. Ranking of the potential compound was performed using dock score and MMGBSA based free energy. The study reported that the docking score of aphanorphine (−6.010 Kcalmol^−1^) and alpha-dimorphecolic acid (ADMA) (−6.574 Kcalmol^−1^) showed significant role with respect to the reported potential inhibitor PC190723 (−4.135 Kcalmol^−1^). A 100 ns MD simulation infers that Fts-Z ADMA complex has a stable conformation throughout the progress of the simulation. Both the compounds, i.e., ADMA and Aphanorphine, were further considered for In-vitro validation by performing anti-bacterial studies against *B. subtilis* by agar well diffusion method. The results obtained through In-vitro studies confirm that ADMA, a small molecule of cyanobacterial origin, is a potential compound with an antibacterial activity that may act by inhibiting the novel target Fts-Z and could be a great drug candidate for antibiotic development.

## 1. Introduction

Cyanobacteria, or blue-green algae, is one of the largest genera groups of organisms, having over 3000 species from terrestrial, marine, and freshwater systems [1]. They are considered as the largest reservoir of metabolites (over 28,000) contributing to antibacterial [2], antifungal [3], anticoagulant, antiviral [4], antimalarial, antitumor [5] anticancer, anti-inflammatory [6,7,8], antiprotozoal, anti-tuberculosis [9], algicidal, and enzyme inhibition activities [10]. The potential of cyanobacterial compounds is endless, and hence scientists and pharmaceutical industries are exploring the potential bioactive molecules for suitable applications [11].

Antimicrobial resistance (AMR) is becoming a grave challenge in the health care sector, since the pathogenic bacteria have ceased to respond to several broad-spectrum antibiotics [10,11]. This has been reported to cause medical complications after surgery [12], and even death in some cases [13,14]. A report published by The Organization for Economic Co-operation and Development (OECD) in the year 2018 speculates that, amongst the many developed nations, 2.4 million individuals may die due to infections with antibiotic-resistant microorganisms in the next 30 years [15,16].

The mechanism of antibiotic resistance, whether intrinsic or acquired, can be subdivided into four classes: (a) modification of antibiotic binding site residues, (b) altering the binding position, (c) destroying the antibiotic through bacterial enzymes, (d) and lowering the antibiotic amount by flushing out from the cell [17,18,19,20,21].

The majority of pathogenic bacteria have three common targets for conventional antibiotics, namely: cell wall biosynthesis to target the cell division machinery, protein synthesis to inhibit the synthesis of essential proteins, and DNA replication/repair that inhibits the growth and proliferation [22,23]. Marston, in 2016, studied various strategies to fight back against AMR, wherein one of the approaches used in the study was to use the untapped potential of natural products, such as marine microbes living in extreme conditions, as a source of antibiotic discovery [24].

Bacterial cell division machinery is a potential pathway for antibiotic treatment [25]. The filamentous thermosensitive sensitive mutant Z protein (Fts-Z) is a conserved 40 kDa protein, considered as a homolog of prokaryotic tubulin, which plays a central role in bacterial cell division [26]. The Fts-Z has an enzymatic N-terminal domain and a flexible long C-terminal domain [27], and its impairment leads to cell lysis via filamentation in bacilli and ballooning in cocci [28,29,30]. It forms a highly dynamic Z-ring at the center of the cell undergoing a division and recruits other accessory proteins, which are involved in bacterial cytokinesis, which is considered essential for bacterial viability being conserved in most bacteria and defines the plane of cell division [31]. In the absence of a proper Fts-Z assembly, functional Z-ring formation does not occur [32,33]. The bacterial cell division thus becomes interrupted irrespective of normal DNA replication and nucleoid segregation, and the cell continues to elongate, resulting in a filamentous appearance ultimately leading to cell death [34].

**Figure 1:**
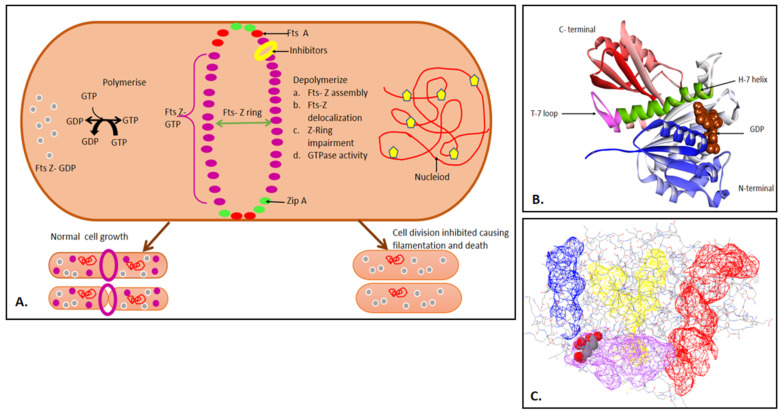
(**A**) An illustration representing the role of Fts-Z in the Z-ring assembly pathway, emphasizing normal and interrupted cell division in bacteria; (**B**) Structure of Fts-Z protein from S. aureus (PDB ID-2VXY) in ribbons bound to GDP (in brown CPK). The T7 loop is in pink and α-H7 helix is in green. The blue-colored portion is the N-terminal and the green portion is the C-terminal. The figure was visualized in BIOVIA Discovery studio visualizer v21.1; (**C**) The binding sites present on the Fts-Z protein. (Figure generated through PDBSUM) [37].

Absence of Fts-Z or any of its functional homologues is not found in mitochondria of higher eukaryotes, thus the inhibitor may not affect any of the host functions. The pathway and biochemical activities have also been studied to a greater extent [35,36]. Figure 1A shows the mechanism used by the cytoskeleton for synthesizing the *B. subtilis* cell wall. Treadmilling occurs between Fts-Z filaments, and peptidoglycan synthesis occurs. Fts-Z monomers along with GTP hydrolysis forms GDP bound Fts-Z monomers, which are then lost from the other side, as shown in brown arrows. Figure 1B shows the domains of Fts-Z protein. It has an unstructured, short, and less conserved N^−^ terminal; a conserved core which is comprised of major portions of the N^−^ and C^−^ terminal domain and GTP binding site joining alpha helix (H7); and a C^−^ terminal region which varies. Figure 1C shows the major binding sites on the Fts-Z protein in blue, yellow, red, and purple. The figure was generated through the PDB Sum Generate tool. There were four binding sites found on the protein; the first cavity (in red) was the biggest, with a cavity volume of 3176.72 Å^3^, yet was unoccupied. The second cavity (in purple) was the GTP binding site, having a volume of 1950.75 Å^3^. The third cavity (in yellow) had a volume of 1609.45 Å^3^, and the fourth cavity (in blue) had a volume of 1024.95 Å^3^.

Certain known inhibitors of Fts-Z protein for Gram-positive bacteria are viriditoxin [12], 2-hydroxy5-benzylisouvarinol-B, and dichamanetin [26]. Sanguinarine and zantrin are used for other pathogenic bacteria [38,39]. Compounds Z5 amongst Zantrins (Z1-Z5) and a 4-Aminofurazan derivative, A189 inhibiting GTPase activity [40], and sanguarins disturb the ring formation and can be toxic as a target to mammalian cells [41,42,43].

Haydon, in his studies, validated Fts-Z as a target for antibacterial intervention with a class of small synthetic compounds. Curcumin is also reported to target the Fts-Z protein from *B. subtilis* (PDB ID-2VAM) and E. coli (homology modelled Fts-Z receptor) with the binding affinities of −17.55 Kcalmol^−1^ and −18.84 Kcalmol^−1^, respectively, in an In-silico study [44].

*B. subtilis* is a widely studied bacterium because of its adaptable metabolism and remarkable physiological characters, and it is easy to cultivate on low-cost substrates. In addition to these, it has an efficient protein secretion system wherein it has three pathways, namely the ATP-binding cassette (ABC) transporters, the twin-arginine translocation pathway (Tat), and the general protein secretion pathway (Sec). Chiefly, the Sec-SRP (Signal Recognition Particle) cooperation route is responsible for the transportation of the greatest amount of protein exported. Additionally, this organism is considered in the ‘Generally Recognized as Safe (GRAS) category’ over other Gram-positive and negative organisms, and thus is the target organism in most research [45,46]. In the present research, 165 bioactive compounds of cyanobacterial origin were screened, and antibacterial activity is reported against the Fts-Z protein of *B. subtilis*. The study included a target-based protein-ligand docking using the Glide module of the Maestro v-2019-2 suite with standard precision (SP) and extra precision (XP) docking, followed by molecular dynamic simulation of the ligand-Fts-Z complex using Desmond module. The study was further supported by In-vitro experiments such as agar well diffusion bioassay and estimation of minimum inhibitory concentration (MIC). The lead molecule in the study was identified as alpha dimorphecolic acid (ADMA), which is an endogenous fatty acid also found from different sources of plants [47,48,49], marine fungi [50], and sea algae [51], and has been reported to be synthesized by various researchers [52].

## 2. Results

### 2.1. Preparation of Receptor and Its Binding Site

The 3D structure was analyzed through PDB-SUM, which showed that the protein consists of 14 α-helices, 12 helix–helix interacts, 17 β turns, and 1 γ turn [53]. The structure was assessed in the protein preparation wizard of the Maestro v-2019-2 suite which provided the backbone dihedral angles (ϕ and ψ) based on statistical distribution of amino acids through the Ramachandran plot. It showed maximum residues to be in the allowed region, asserting that the receptor structure was in better condition to proceed with virtual screening studies. The structure was minimized and used for further docking procedures.

**Figure 2:**
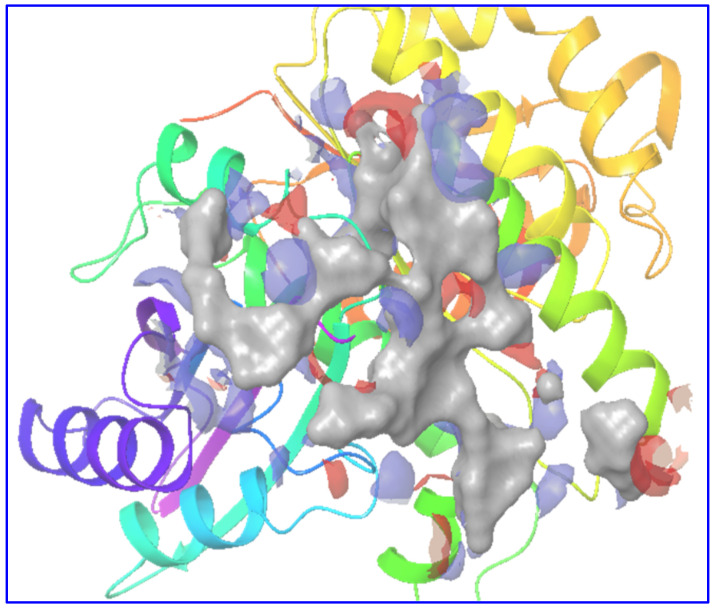
The figure shows the grid formed around the receptor Fts-Z (PDB ID 2VXY), taking the site map tool’s predictions. Site points (presented on grey surface) for the potential binding site (site-1). The site score was 0.911 with a size of 791. Dscore- was 1.028, Volume—558.061, HB don./acc.—1.401, Hydrophilic—0.185, Hydrophobic—0.464.

The site map tool provided the five best sites depending on their hydrophobicity, hydrophilicity, hydrogen bond donating, and accepting capabilities. The literature claimed that residues Arg 29 and Arg 191 are important for binding with the ligands and bond formation [27,54]. Amongst the top five sites, a site that contained Arg 29 and Arg 191 residues was convincing to use for ligand docking. Accordingly, a grid was formed using these site points provided by the site map tool. A figure appended below demonstrates the predicted potential binding site (site-1) on the protein model (Figure 2).

### 2.2. Virtual Screening and Molecular Docking

**Figure 3:**
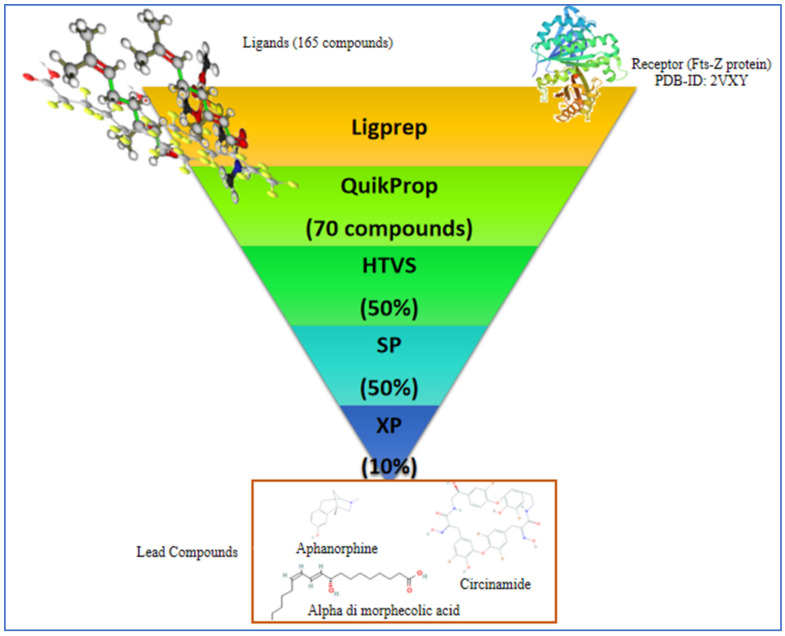
Schematic figure showing steps followed during virtual screening of compounds.

Virtual screening of the compounds was performed by the VSW module of the Maestro suite (2019.2 version), which included LigPrep for preparation of ligands, QikProp for filtering out ligands on the basis of physicochemical properties, and Glide docking at three precision levels, i.e., HTVS, SP, and XP. HTVS and SP modes were used to narrow down the large dataset of ligands, and XP mode gave many accurate results in comparison to the above modes. Once the correct form of receptor and ligand was ensured, the receptor grid was generated based on literature review and site map results. Figure 3 is a flow diagram to show the steps followed during docking.

Glide initially places the center of the ligand at different grid points of 1 Å and rotates them around three Euler angles. Then, filtration is conducted based on grid-based force field evaluation and then further refinement of docking solutions is completed by torsional and rigid body ligand movements. The overall final energy assessment is completed with Glide Score and, for each ligand, a final best posture is generated as the output. The Glide Score is calculated by summing ligand–protein interaction energy, hydrophobic interactions, π–π stacking interactions, hydrogen bonds, internal energy, RMSD (root mean sq. deviation of protein before and after docking), and desolvation scores.

G Score = a × vdW + b × Coul + Lipo + Hbond + Metal + RotB + Site(1)

where vdW = van der Waal energy; Coul = C energy; Lipo = Lipophilic contact term; HBond = hydrogen-bonding term; Metal = metal-binding term; RotB = penalty for freezing rotatable bonds; Site = polar interactions at the active site; and coefficients of vdW and Coul are a = 0.065 and b = 0.130 [55].

**Figure 4:**
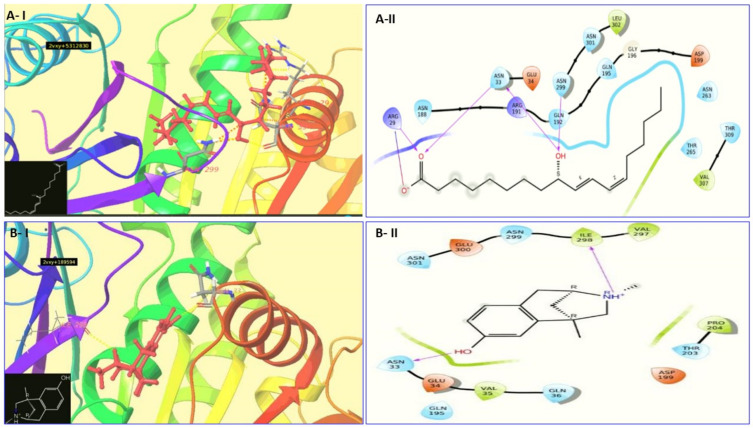

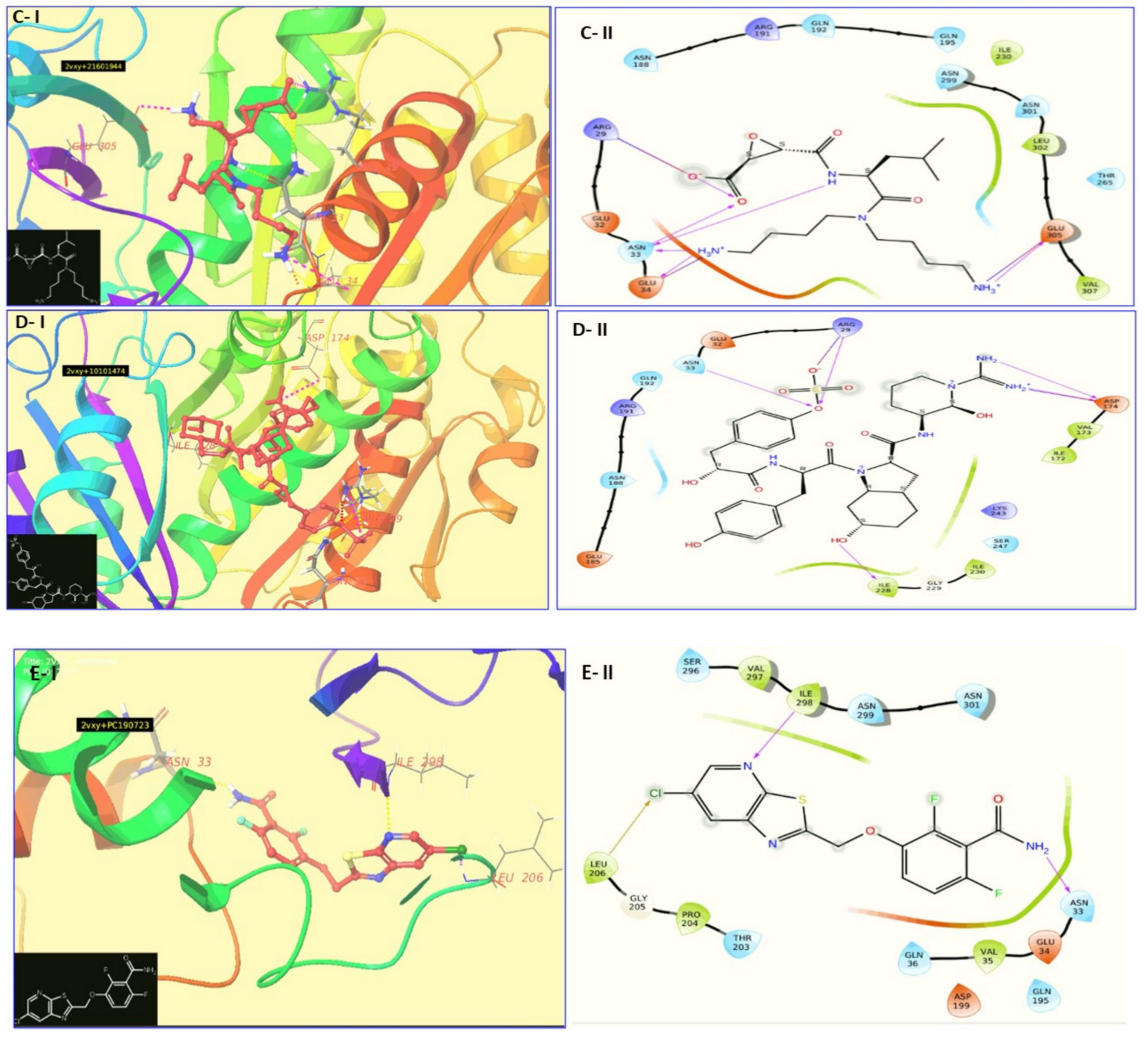
The 2D and 3D diagrams of docking poses. (**A-I**) 3D interaction diagram of Alpha dimorphecolic acid (5312830) with Fts-Z protein.; (**A-II**) 2D interaction diagram of Alpha dimorphecolic acid (5312830) with Fts-Z protein.; (**B-I**) 3D interaction diagram of Aphanorphine (189594) with Fts-Z protein. (**B-II**) 2D interaction diagram of Aphanorphine (189594) with Fts-Z protein.; (**C-I**) 3D interaction diagram of Circinamide (21601944) with Fts-Z protein. (**C-II**) 2D interaction diagram of Circinamide (21601944) with Fts-Z protein.; (**D-I**) 3D interaction diagram of Aeruginosin 102 A (10101474) with Fts-Z protein. (**D-II**) 2D interaction diagram of Aeruginosin 102 A (10101474) with Fts-Z protein.; (**E-I**) 3D interaction diagram of PC190723 (25016417) with Fts-Z protein. (**E-II**) interaction diagram of PC190723 (25016417) with Fts-Z protein.

The output of Glide was exported to PDB ID format and further studies of interactions were conducted in the Maestro v-2019-2 interface and Biovia Discovery studio visualizer (Dassault Systèmes, 2020). The summary of the docking results is shown in Table 1, and Figure 4 shows 2D and 3D interaction diagrams.

**Figure 5:**
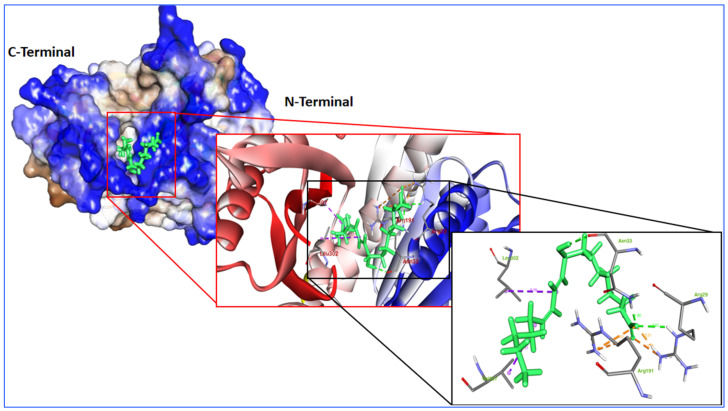
Surface is added to Fts-Z protein (PDB ID-2VXY) on the basis of hydrophobicity, and ADMA compound (in green) is shown to be docked in the binding cavity and another zoom in figure is shown for interacting residues of the protein with ligand. Further Zoom in view of the ADMA compound and interacting residues from binding cavity. The visualization was done through Biovia Discovery Studio Visualizer.

ADMA is the cyanobacteria-based molecule which has been found to inhibit Fts-Z receptor through In-silico techniques. The docking results of our study is in favor of previous studies that Arg 29 and Arg 191 for the Fts-Z protein are crucial and active sites for ligand binding. Figure 5 show zoom in view of the ADMA compound bound to receptor along with stick representation (in green) of the interacting residues.

### 2.3. ADME Analysis Using Qik Prop Module

The compounds were screened with Lipinski Rule of Five to assess their pharmacological and drug-likeliness properties, and those which qualified RO5 were subjected to HTVS, SP, and XP mode of docking [56]. Some other physicochemical parameters were taken into consideration, such as predicted octanol/gas partition coefficient (log Poct), predicted water/gas partition coefficient (logPo/w), aqueous solubility prediction (log S), the IC50 value for the blockage of HERG K^+^ channels (log HERG), gut–blood barrier (log Caco), the blood–brain barrier permeability (log BB), essential for passage of drugs to the brain, (log Khsa) predicting binding to human serum albumin, (log Kp) predicting skin permeability, and percentage of human oral absorption (PHOA). The acceptable range of these descriptors was used for obtaining druggable molecules which were taken further for experiments and analysis. The following Table 2 depicts the values of hit molecules from the QikProp module.

The table shows the phytochemical properties of compounds that had good docking affinity and it is evident that all the values are in the acceptable range for the ADMA which indicates that it can be taken for further experiments and has the caliber to become a good drug candidate.

### 2.4. Molecular Dynamics and Simulation

**Figure 6:**
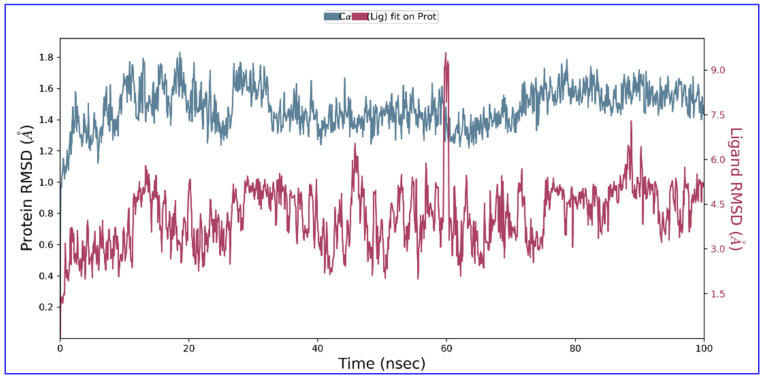
The RMSD (in Å) of Fts-Z protein (PDB ID- 2VXY) with ADMA (ligand) compound during 100 ns MD trajectory. The fluctuations of both the ligand and the receptor were in an acceptable range. Fts-Z protein (in green) fluctuation was within 2 Å, achieving stability towards the end of the simulation, and that of ligand (in maroon) with respect to protein and its binding pocket was also not fluctuating significantly showing a stabler confirmation.

The findings of molecular docking studies, although quite indicative of the actual scenario of protein–ligand binding, yet MD simulations provided in-depth inside of interaction revealing even the smallest variations. Thus, an MD simulation study was done for receptor Fts-Z (PDB ID-2VXY) complexed with ADMA (PubChem ID: 5312830) within the solvent system for 100 nanoseconds (ns) to find the atomic details of molecular interactions. Through the Root Mean Square Deviation (RMSD) analysis of the C alpha atoms, it was observed that the complex stabilizes after nearly 20 nanoseconds and remains steady thereafter with slight perturbance near 60 ns and 90 ns. RMSD between this point displayed a deviation of 0.2 Å which might occur due to reorientation of the ligand at the binding cavity. RMSD concerning its starting position increased to 1.8 Å for the first 20 ns and, later than that point, became more stabilized (Figure 6 attached below). All these RMS deviations are within the acceptable region. These values indicated that stability was there in the receptor–ligand complex throughout the progress of the simulation.

**Figure 7:**
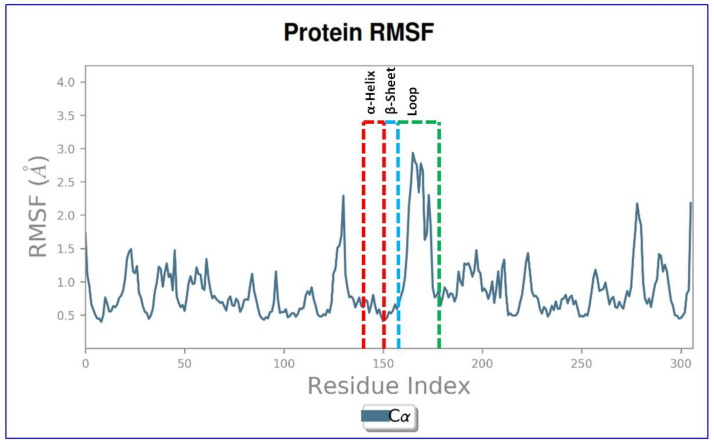
The Root Mean Square Fluctuation (RMSF) of Fts-Z protein (PDB ID-2VXY) throughout 100 ns molecular dynamic simulations showing local fluctuations along with the receptor.

The Root Mean Square Fluctuation (RMSF) property shows the average deviation of the receptor relative to the reference position. From Figure 7 it is evident that the RMSF value of the protein backbone residues is in the range of 0.5–2.5 Å. In the RMSF plot, the fluctuations of top peaks have been shown with respect to main backbone positions from residues Pro 135 to Leu 145 (RMSF-2.5 Å), Thr 160 to Leu 170 (RMSF-3.0 Å), and Leu 270 to Asp 280 (RMSF-2.0 Å) of receptor–ligand complex. Between 140–165 residues predominantly conformed into specific secondary structures. α-helices conformed between 140–150 residues, while from 150–160 both β-sheets and α-helices are predominant.

**Figure 8:**
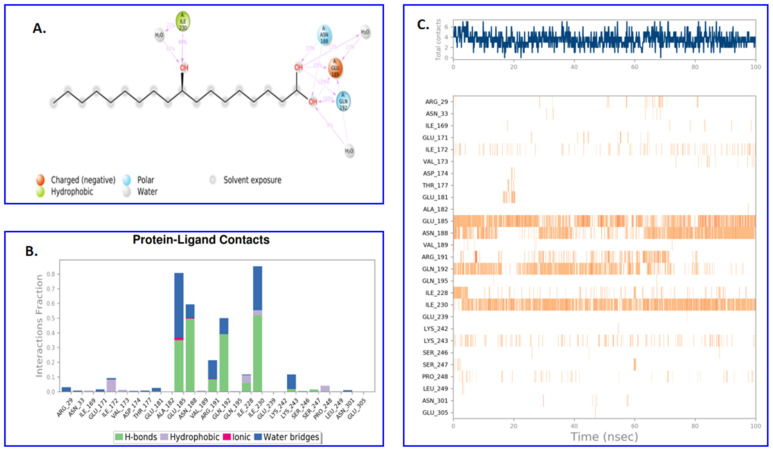
(**A**) Two-dimensional diagram of contacts made more than 9% of simulation time between ADMA (ligand) and Fts-Z (PDB ID-2VXY) (receptor) in 100-ns molecular dynamics. Gln 192 showed a contact time of 110% because it had multiple interactions of a single type with ADMA atoms. (**B**) The plot represents the interaction of the ADMA with 2VXY residues in simulation. (**C**) A timeline representation of residues interacting with the ligand along with density in each trajectory frame.

During MD simulation, residues Ala 182, Glu 185, Val 189, and Ile 228 played an important role at the binding site, and thus are vital for the stabilization of lead compound ADMA (PubChem ID: 5312830) in the binding cavity, as shown in the Figure 8.

The Ligand–Protein Contacts plot obtained after MD simulation also showed that Gln 192 was forming polar contacts and can be attributed to being responsible for stable conformation of the ADMA compound, as it participated 110% of the simulation time (also shown in Figure 8A). Similarly, Asn 188 was also responsible for forming polar contacts with the ligand at the two -OH sites for 33% and 15% of the simulation time at the binding site. Hydrogen bond networks were shown to be forming at the first -OH sites, with residues Ile 230 in 49% of MD simulation time (Figure 8B). Ile 230 also forms a hydrophobic contact. No intramolecular hydrogen bonding was seen. Figure 8C shows the number of residues contacting with ligand and the densities are shown in color dependent manner; the darker the orange color indicates higher number of contacts on that frame. The contacts are grouped in four categories: Hydrogen bonds, hydrophobic bonds, water bridges, and ionic bonds. The top panel of Figure 8C shows total contacts of ADMA compound in the binding site of Fts-Z protein throughout the trajectory and the bottom panel shows the per residue interaction. Residues Glu 185, Asn 188, Gln 192, and Ile 230 were found to interact in almost all frames. Residues Arg 29 and Arg191, although, had lesser interactions. Although previous studies have reported the important of Arg 29 and Arg 191 in the binding cavity of the Fts-Z [27,54], in our study during the analysis of ADMA docking pose revealed an interaction with Arg29; however, it could not be sustained during the course of MD simulation. MD simulation calculates the dynamic behavior of protein–ligand interaction in presence of water and ions. However, the dynamic environment is absent in docking. Therefore, the lack of dynamic environment in molecular docking showed a significant interaction of the residue Arg29 which might have lost during the simulation.

**Figure 9:**
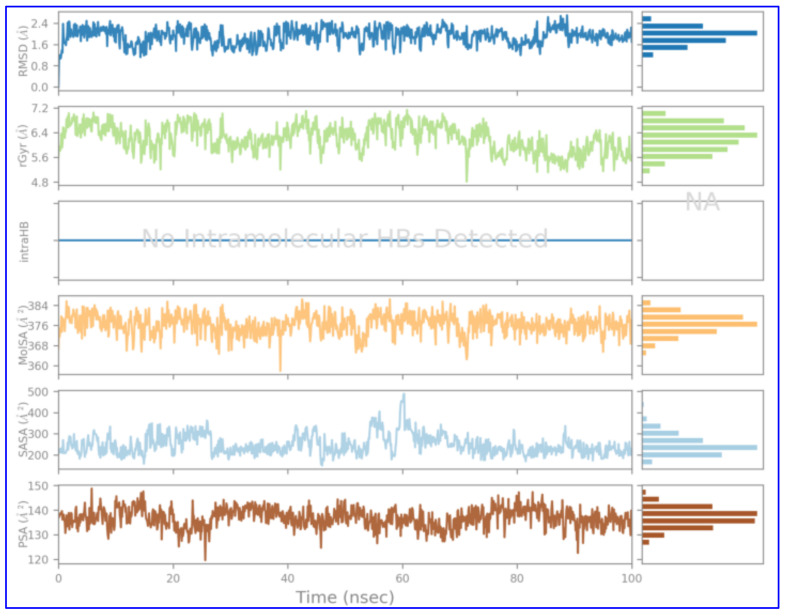
Ligand properties—Root mean square deviation (RMSD) of the ligand with respect to the reference conformation; rGyr (Measure of Extendedness of ligand); intraHB; MolSA; SASA; PSA.2.5. In-vitro Validation of Lead Compound.

The radius of gyration (ROG) predicts the firmness of protein structure [57]. The Fts-Z and ADMA complex was studied for understanding the consequence of ADMA on the compactness of the Fts-Z receptor, and it was found to be a little stretched by retaining an average of 6.05 Å within a range of 4.8–7.1 Å during the simulation. The ADMA also displayed a high solvent-accessible surface area (SASA), polar surface area (PSA), and molecular surface area (MolSA). These all parameters were in favor of the stability of the Fts-Z and ADMA complex in simulation. Figure 9 shows the RMSD, Radius of Gyration, Intra Hydrogen bonds, Molecular surface area, Solvent accessible surface area, Polar surface area.

Initial In-vitro validation of methanolic extract of *Oscillatoria* sp. (100 µgmL^−1^) was done using the agar well diffusion method. A significant inhibition zone of 16.14 mm was obtained by methanolic extract of *Oscillatoria* sp. was against *B. subtilis*-MTCC441. Further, a concentration of 5 µgmL^−1^ of ADMA and aphanorphine was also tested against the same bacterial strain.

Results of the antibacterial evaluation are summarized in Table 3 and Table 4. The lead compound, ADMA exhibited a potential antibacterial activity (Zone of inhibition 23.12 mm) against *B. subtilis*. However, aphanorphine did not exhibit any sort of antibacterial activity in terms of zone of inhibition against *B. subtilis*. A known inhibitor, i.e., Polymyxin B-sulphate, at a concentration of 5 µgmL^−1^, was also found to be inhibitory (21.33 mm) against *B. subtilis*-MTCC441. The MIC (Minimum inhibitory concentration) of ADMA, Polymyxin B-sulphate, and methanolic extract of *Oscillatoria* against *B. subtilis* were determined to be 512 µgmL^−1^, 256 µgmL^−1^, and 1024 µgmL^−1^, respectively [47].

**Figure 10:**
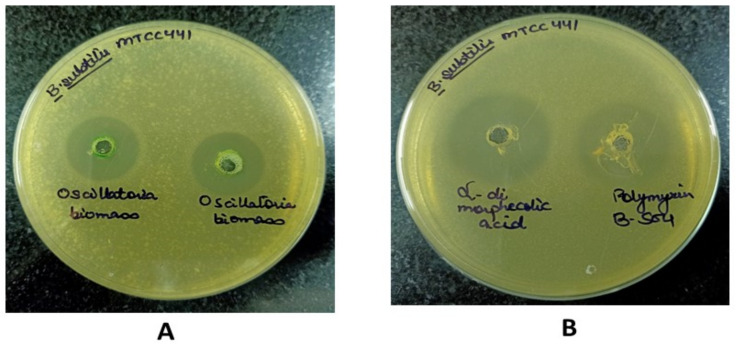
Zone of inhibition against *Bacillus subtilis* (MTCC 441): Muller Hinton Agar plate, (**A**) well size: 16.14 mm for Methanolic extract of *Oscillatoria* biomass (100 µgmL^−1^). (**B**) 23.12 mm for Alpha dimorphecolic acid (5 µgmL^−1^) 21.33 mm for Polymyxin B-sulphate (5 µgmL^−1^). Zone size in mm was recorded after 24 h of incubation time at 37 °C.

**Figure 11:**
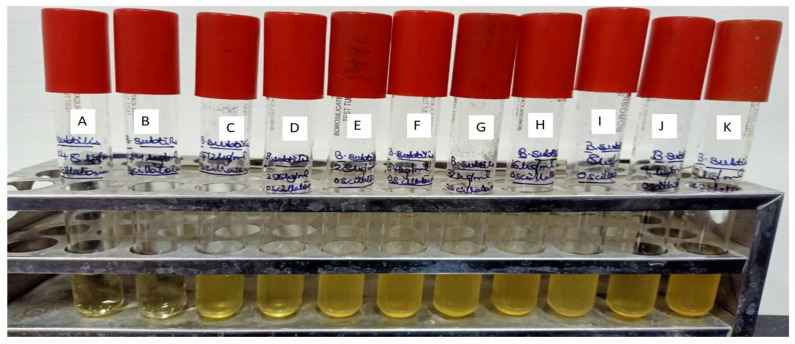
Minimum inhibitory concentration of Methanolic extract of *Oscillatoria* against *Bacillus subtilis* (MTCC 441) Muller Hinton agar broth tubes A to K dilution range from 1024 to 1 µgmL^−1^. A—1024 µgmL^−1^, B—512 µgmL^−1^, C—256 µgmL^−1^, D—128 µgmL^−1^, E—64 µgmL^−1^, F—32 µgmL^−1^, G—16 µgmL^−1^, H—8 µgmL^−1^, I—4 µgmL^−1^, J—2 µgmL^−1^, K—1 µgmL^−1^.

**Figure 12:**
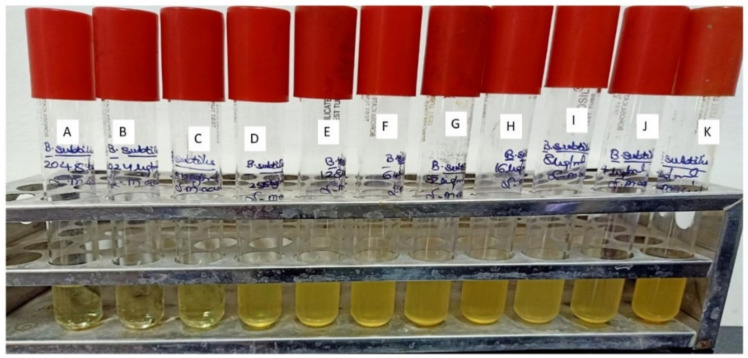
Minimum inhibitory concentration of Alpha dimorphecolic acid against *Bacillus subtilis* (MTCC 441) Muller Hinton agar broth tubes A to K dilution range from 1024 to 1 µgmL^−1^. A—1024 µgmL^−1^, B—512 µgmL^−1^, C—256 µgmL^−1^, D—128 µgmL^−1^, E—64 µgmL^−1^, F—32 µgmL^−1^, G—16 µgmL^−1^, H—8 µgmL^−1^, I—4 µgmL^−1^, J—2 µgmL^−1^, K—1 µgmL^−1^.

**Figure 13:**
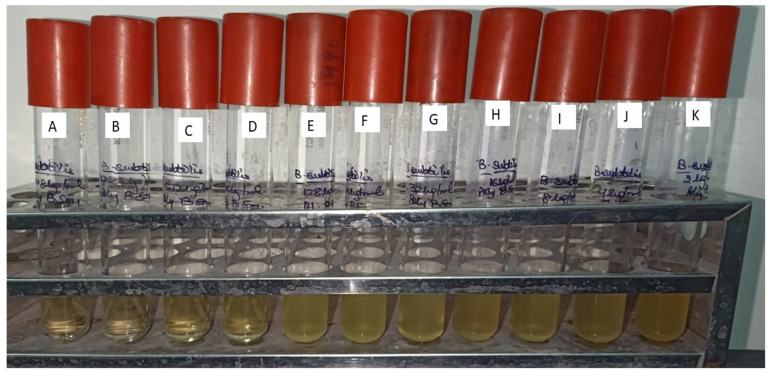
Minimum inhibitory concentration of Polymyxin B sulphate against *Bacillus subtilis* (MTCC 441) Muller Hinton agar broth tubes A to K dilution range from 1024 to 1 µgmL^−1^. A—1024 µgmL^−1^, B—512 µgmL^−1^, C—256 µgmL^−1^, D—128 µgmL^−1^, E—64 µgmL^−1^, F—32 µgmL^−1^, G—16 µgmL^−1^, H—8 µgmL^−1^, I—4 µgmL^−1^, J—2 µgmL^−1^, K—1 µgmL^−1^.

In Figure 10, Figure 11, Figure 12 and Figure 13, for the methanolic extract of Oscillatoria, ADMA, and Polymyxin B sulfate. Figure 11 is showing the In-vitro validation through agar well diffusion technique and MIC calculation.

## 3. Discussion

The drug discovery and development sector are putting immense efforts to discover potential drug candidates which can target the cell division machinery of bacterial pathogens. In present research, In-silico molecular docking studies of 165 compounds of cyanobacterial origin were carried out against the receptor Fts-Z. Among them, ADMA was identified as a top ranked compound with docking score –6.574 Kcalmol^−1^. The study showed that ADMA binds to the hydrophobic cleft formed between the C-terminal domain and α7 helix. The known inhibitor was PC190723 which had a docking score of –4.135 Kcalmol^−1^ and interacting residues were Asn 33, Gln 192, Ile 228, Ser 247, Ile 298 forming hydrogen bonds, Ile 172, Ile 228 forming Pi-alkyl bond, Ile 228 forming halogen bond. ADMA had Asn 33 and Asn 299 forming hydrogen bonds, Arg 29, Arg 184, Arg 191 forming salt bridges, Leu 302 and Val 307 forming Alkyl bonds. Our findings suggest that ADMA may be a potential inhibitor of Fts-Z protein.

A study on inhibition of Fts-Z with compounds Coupene, Proximadiol, and Menthone had a docking score of −6.2 Kcalmol^−1^, −6 Kcalmol^−1^ and −5.7 Kcalmol^−1^, which are lower than docking score of ADMA dealt in our finding [8]. Another study on verticiol as a Fts- Z inhibitor exhibits the docking score of −4.72 Kcalmol^−1^ [58]. These compounds had binding affinity near to ADMA reported in our study. So, it is expected that ADMA may have potent Fts-Z protein inhibition though this can be a future aspect of this study. The qualitative experiments performed along with In-silico results provide indications for the same.

The MMGBSA values for ADMA were −44.12 Kcalmol^−1^ and for PC190723 was −53.36 Kcalmol^−1^. The ADMA—Fts-Z complex was stable in 100 ns molecular dynamic simulations. MMGBSA represents only one of the free-energy calculation techniques and that Linear Response Approximation (LRA) method also yielded reasonable binding free energies for curcumin. Therefore, similarly, the LRA method could also provide reasonable binding energy of ADMA with Fts-Z, as reported elsewhere [59].

Some other compounds were also explored to target the Fts-Z receptor. For curcumin, as reported by Haydon et al., binding affinity was −17.55 Kcalmol^−1^ and residues of Fts-Z that were in contact with Curcumin were Gly 21, Gly 22, Gly 72 and Thr 133, Asn 166 forming hydrogen bond and Glu 20, Leu 69, Gly 70, Ala73, Gly 104, Thr 109, Pro 135, Glu 139, Arg 143, Ala 186, and Asp 187 forming hydrophobic bonds in case of *B. subtilis* [60]. Another group of scientists exploring the 180 endophytic fungal strains from Chinese medicinal plant *Panax notoginseng* found compound Brasiliamide J-a and Brasiliamide J-b from species *Penicillium janthinellum* to be a potential inhibitor of Fts-Z against *B. subtilis* (PDB ID-2VXY), with a binding affinity of −111 Kcalmol^−1^ forming H^−^ bonds with residues Glu 139 and Gly 107 and hydrophobic bonds Asn 166, Gly 104, Gly 21, and Asn 25 for compound Brasiliamide J-a and −122 Kcalmol^−1^ forming H^−^ bonds with Asn 25 and hydrophobic bonds with Met 105, Glu 139, and Phe 183 for compound Brasiliamide J-b and against *S.aureus* (PDB ID-3VOB) [61]. Further, an In-silico study revealed that quinazoline derivatives, Zantrin Z3 and ZZ3, bind *Mycobacterium tuberculosis* Fts-Z protein (PDB ID- 1RQ7-chain A) near the H6/H7 loop and do not affect hydrolysis of GTP, but they are efficient against the aggregation of Fts-Z [58]. Ballu and co-workers performed In-silico receptor-based docking experiments and 3D QSAR using CoMFA and CoMSIA methods to understand binding interactions of 42 molecules (oxazole-benzamide derivatives and aryl alkoxy benzamide derivatives) with the receptor (PDB ID- 3VOB) and highlighted the role of residues Val 207, Leu 209, and Asn 263 in the binding of inhibitors to Fts-Z protein [62]. In a recent study by Somma and coworkers, it was reported that the competitive action mechanism of natural peptide Temporin L against modeled Fts-Z protein from *P. aeruginosa* (PDB ID-2VAW) through docking studies showed the involvement of Phe and Trp residues at the C-terminus of the peptide against Gly 19, Gly 20, Gly 21, Gly105, Gly 106, Gly 107, Ala 72, Leu 178, Asp 45, and Asn 24, and Phe 182 of protein Fts-Z along with side chain of Arg of peptide forming a salt bridge with Glu 138 of protein Fts-Z and validated this further by binding and enzymatic assays. A group of scientists performed rigid protein docking with a library of pyrimidine-substituted quinuclidines compounds against Fts-Z protein (PDB ID-1W5B), and then further analyzed the binding of eight different bacterial proteins (PDB ID-1OFU, 1RLU, 1W5B, 1W5F, 2R6R, 2R75, 2VAP, 2VXY) against the lead compounds through induced fit docking method and found that Quinuclidine 12 to be useful for both gram-positive and gram-negative bacteria [63].

The MIC values of ADMA in our work were observed to be 512 µgmL^−1^ and the zone of inhibition of 23.12mm at 5 µgmL^−1^. The results are also in correlation with antibacterial activity against *B. subtilis* SBUG14 where ADMA gave a zone of inhibition of 3.5 mm at 100 µgmL^−1^ [64]. A team of researchers from AIIMS, New Delhi, India, in their work showed that amongst the FDA-approved drugs, benzethonium chloride gave a MIC value of 8 µgmL^−1^ for *Salmonella Typhi* (ATCC 19430), 1 µgmL^−1^ for *Staphylococcus aureus* (ATCC 43300) and 12 µgmL^−1^ for *Escherichia coli* (ATCC 25922) [65]. Another group of researchers prepared trisubstituted oxazoles which showed the high potencies against *S. aureus* with an MIC of 0.03 µgmL^−1^ [66].

Here we report the identification of Fts-Z as intracellular targets for cyanobacterial compound ADMA, indicating its pathway for antibacterial activity. This fatty acid compound binds, Fts-Z of *B. subtilis* in the In-silico protein ligand docking and in In-vitro experiments the compound was found to inhibit bacterial growth in the micromolar range, and which might be due to perturbation of the Z-ring assembly. Aphanorphine, which is an analgesic alkaloid by nature was the second hit compound of the study with a binding affinity of –6.010 Kcalmol^−1^. The In-vitro activity of this compound was nil so was not taken further. The absence of activity may be attributed to the methods and types of extracts used during In-vitro validation. Authors will keep this point for future prospects.

## 4. Materials and Methods

### 4.1. Target and Ligands Retrieval

Nearly 165 cyanobacterial compounds belonging to genera of *Anabaena*, *Calothrix*, *Cylindrospermum*, *Dichothrix, Fischerella*, *Hapalosiphon*, *Hormothamnion*, *Hyella*, *Lanthella, Leptolyngbya*, *Lyngbya*, *Microcoleous*, *Microcystis*, *Nadularia, Nostoc*, *Oscillatoria*, *Schizothrix*, *Scytonemma*, *Spirulina*, *Symploca*, *Synechococcus*, *Tolypothrix*, and *Trididemnum*. All cyanobacterial compounds belonging to lipopeptides, macrolides, polyketides, poly phenyl ether, cyclic peptide, indole alkaloid, cyclophane, depsipeptide, hexapeptides, diterpenoid, diterpenes, alkaloid, peptide, porphinoids, fatty acid, glycosides, and phenol derivatives were compiled and then, through extensive literature review data for habitat, reported activity along with target organism was noted. Then, the structures of corresponding compounds were retrieved from PubChem (https://pubchem.ncbi.nlm.nih.gov/; accessed on 23 January 2021) in SDF format.

There were more than 10 crystal structures available in Protein Data Bank for Fts-Z protein of *B. subtilis*. The selection of protein is conducted as per Deller and Rupp, 2015. The structure with PDB ID- 2VXY is an X-ray resolved structure that has a citrate ion bound to it with a potassium metal (ion or atom); the structure has a resolution of 1.7 Å that was selected for this study. The secondary structure of the receptor was analyzed through PDB-SUM [67].

### 4.2. Preparation of Ligands and ADME Analysis

All the ligands in the SDF format were imported in the LigPrep module of Maestro v-2019-2 Suite, 9.2v (LigPrep (2019) New York, NY, USA; Schrödinger, LLC). The compounds were ionized and desalted at neutral pH 7 ± 2.0 with an ionizer. Compounds were then corrected for bond orders and energy was minimized using Optimized Potentials for Liquid Simulations (OPLS) 2005 force field for generating low energy conformations for each ligand. This step corrected the three-dimensional structure of the compounds in terms of bond order and ionization states and generated stereoisomers with possible ionization states.

Qik Prop module of Maestro v-2019-2 suite was used to predict the physical and pharmaceutical descriptors such as adsorption, distribution, metabolism, and excretion (ADME) of the compounds and provided the minimum and maximum point for molecular property comparison with those of 95% known drugs. The compounds screened by multi-stage docking study were further quantitatively assessed for their pharmacokinetic properties using ADME analysis. All the hit molecules were neutralized and their SDF format was used as an input for the analysis [37].

### 4.3. Preparation of Receptor and Its Binding Site

Receptor protein was prepared through the Protein Preparation Wizard of Maestro v-2019-2 9.2v (Schrodinger Inc., USA), which helped in correcting any pre-existing problems in the molecule. The water molecules were removed beyond 5 Å from hetero groups and hydrogen atoms were added. The missing side chains were added using the Prime module [68,69]. The geometry refinement was performed by energy minimization using OPLS 2005 force field until the RMSD value for heavy atoms reached 0.3 Å and verified through the Ramachandran plot.

The sitemap program of the Maestro v-2019-2 suite was used to predict the binding site using physical descriptors such as degree of enclosure, exposure, size, hydrogen bonding possibilities, tightness, hydrophilic, and hydrophobic site points, based on protein characteristics which are used for generating grid for docking. The OPLS-2005 force field was employed to generate pockets and to calculate charge density [70,71,72]. The site containing Arg191 and Arg 29 residues was selected amongst the output of the site map tool, as reported in previous studies. The binding grid was formed by the Glide module that uses the force field information, resulting in an outer box and an inner box indicating possible positions for the ligand center and the total space for all ligand atoms.

### 4.4. Virtual Screening and Molecular Docking

Before docking, ligands were checked for drug likeliness through implementing Rule of Five (RO5) [56] through Virtual Screen Workflow (VSW) module wizard and also implementing the findings of the QikProp module. The ligands containing reactive functional groups were removed.

The output for VSW workflow was set to the following attributes: High Throughput Virtual Screening (HTVS) was set to give 50% of the total, which went for Standard Precision (SP) to give 50% again. These were fed to Extra Precision (XP), giving 10% compounds, and this was ranked based on Glide score.

Molecular docking of Fts-Z as a receptor protein (PDB ID-2VXY) and cyanobacterial compounds was conducted using the Glide module on the VSW of the Maestro v-2019-2 suit. Glide scores were considered for ranking the compounds [73]. Through the Glide score, the binding energy and force field, along with penalties and accurate results during the docking process, are revealed and the thus potent bioactive molecule is predicted to have Fts-Z inhibitory action [74].

### 4.5. Binding Free Energy Calculation

The binding free energy of the final complexes was calculated using Prime/MM-GBSA in the presence of the OPLS force field in the variable dielectric (VSGB) solvent model [26]. The MM-GBSA (ΔG bind) was estimated in kilocalories per mole through the following equation:

ΔG (bind) = E (complex) − E (protein) − E (ligand)

### 4.6. Molecular Dynamics and Simulation

The MD simulation was carried out using Desmond v3.6, 2013 of the Maestro v-2019-2 suite to analyze the stability of the protein–ligand interaction in a dynamic environment, using OPLS-2005 force field and keeping default parameters protocol [75,76]. For building the simulation environment, three-site rigid water molecules with charges and Lennard–Jones parameters assigned to each of the three atoms (TIP3P) water molecules were filled in an orthorhombic cubic box. Buffering was done at a distance of 10 Å between the edges of the box and receptor atom. Boundary condition box volume was calculated to be 478,000 Å^3^. The system was neutralized by adding Na^+^ and Cl^−^ counter ions. The receptor had 22,073 atoms and 5831 water molecules. Regarding the Molecular dynamic simulations conditions, an isothermal isobaric ensemble (NPT) keeping the temperature 300 K, pressure 1 atm (Standard Atmospheric Pressure), and thermostat relaxation time of 100 ns was used. The system was simulated in the Berendsen NPT ensemble keeping the temperature at 10 K to restrain heavy atoms on solute. Nose-Hoover thermostats and Martynas-Tobias-Klein bar stat methods were followed to maintain the temperature scale and pressure at 300 K and 1 atm respectively during MD simulation. The simulation process was conducted by 15 ns NPT production, and at the interval of every 5 ps, the configurations of each event were collected.

### 4.7. In Vitro Validation of Lead Compound

Agar well diffusion assay and macro broth dilution method were used to validating inhibitory action of Aphanorphine (5 µgmL^−1^) and ADMA (5 µgmL^−1^) along with methanolic extracts (100 µgmL^−1^) of *Oscillatoria* sp. against *B. subtilis* MTCC-441 [47,48,51,52,64,77]. Polymyxin B sulfate (5 µgmL^−1^) was used as a positive control, which is an antibiotic for treating various bacterial infections [77]. All the bacteriological media used in the study were procured from Hi-Media Laboratories, Mumbai, India, and all reagents and chemicals of analytical grades were procured from Sigma Aldrich, Bangalore, India.

## 5. Conclusions

Our study concludes that ADMA is a potential antibacterial candidate against the Fts-Z protein as an exclusive target in the gram-positive *B. subtilis* (MTCC 441) with the binding affinity of −6.574 Kcalmol^−1^ and free energy (MM-GBSA) of −44.12 Kcalmol^−1^. The finding was validated with a zone of inhibition study (23.12 mm) and MIC (512 µgmL^−1^). The MD simulation and In-vitro results are also in concordance with already published reports. In-silico docking, simulation, and other published works convince that ADMA binds to *B subtilis* Fts-Z. The further potential of ADMA and Fts-Z can be revealed by enzymatic bioassay, isothermal calorimetry titration (ICT), and NMR spectroscopy. It may provide new prospects for a natural compound as a potent anti-biotic drug or drug candidate amid growing concern over antibiotic resistance.

## Figures and Tables

**Table 1:** Summary of docking ligand with Fts-Z protein of *B. subtilis*; information about compounds’ source and reported activity with reference.

Bioactive Molecules	Docking Score (Kcalmol^−1^)	MMGBSA-dG Binding (Kcalmol^−1^)	Interactions (Hydrogen Bonds)	∆G (Kcalmol^−1^) [std]	Lig. Efficiency (Kcalmol^−1^)	Compounds Reported in
Alpha di-morphecolic acid (5312830)	−6.574	−44.12	Arg 29, Asn 33, Asn 299	−8.52 [−0.35]	−0.41	Oscillatoria redekei
Aphanorphine (189594)	−6.010	−	Asn 33, Ile 298	−5.45 [−0.35]	−0.36	Aphanizomenon flos aquae
Circinamide (21601944)	−7.366	−	Arg 29, Asn 33, Glu 34, Glu 305	−7.00 [−0.36]	−0.26	Anabeana circinalis
Aeruginosin 102 A (10101474)	−8.666	−	Arg 29, Asn 33, Asp 174, Ile 228	−8.23 [−0.51]	−0.16	Microcyctis viridis
PC 190723 (2501641)	−4.135	−53.36	Asp 33, Leu 206, Ile 298	−7.68 [−0.52]	−0.33	Known Inhibitor

**Table 2:** Estimated physiochemical and pharmacokinetic parameters by QikProp for compounds selected compounds.

Top Hit	Acceptable Range	Compound ID		
		5,312,830	189,594	10,101,474
mol MW (gMol^−1^)	<500	296.4	203.28	386.5
Donor HB	0.0–5.0	1	0	1
Accpt HB	2.0–20.0	2.75	3.50	5.75
QPlog Poct	8.0–35	10.267	12.999	18.416
QPlog Pw	4.0–45.0	5.804	9.579	5.53
QPlog Po/w	−2.0–6.5	1.972	3.654	2.881
QPlog S	−6.5–0.5	−1.751	−5.559	−3.646
QPlog HERG	below −5	−4.01	−5.967	−5.298
QPP Caco	<25 poor and >500 great	729.418	1059	1180
QP LogBB	−3.0–1.2	0.465	−0.83	−0.61
QPP MDCK	<25 poor and >500 great	389.156	526	1275
QPLog Khsa	−1.5–1.5	0.079	0.449	–6.288
QPLog Kp	−8.0–1.0	−4.142	−2.147	−1.761
PHOA	>80% is strong and <25% is weak	89.734	100	100
Ro5	Max., 4.0	0	0	0

**Table 3:** In-vitro validation of lead compound and extract.

Test Organism	Assays	Lead Compound		Known Inhibitor	Oscillatoria Biomass
		Alpha di Morphecolic Acid	Aphanorphine	Polymyxin B-Sulphate	Methanol Extracts
***B. subtilis*** **(MTCC-441)**	Agar Well diffusion assay (Zone of Inhibition in mm)	23.12 ± 0.1	0.0 Nil	21.33 ± 0.47	16.14 ± 0.6
	MIC (µgmL^−1^)	512	NC	256	1024

NC—Not conducted.

**Table 4:** Minimum inhibitory concentration of *Oscillatorial* Methanolic Extract, Alpha dimorphecolic acid, Polymyxin B sulfate, and Aphanorphine against *B. subtilis* (MTCC 441).

	Concentration in (µgmL^−1^)											MIC (µgmL^−1^)
	1024	512	256	128	64	32	16	8	4	2	1	
Methanolic Extract	−	+	+	+	+	+	+	+	+	+	+	1024
Alpha Di Morphecolic Acid	−	−	+	+	+	+	+	+	+	+	+	512
Polymyxin B Sulfate	−	−	−	+	+	+	+	+	+	+	+	256
Aphanorphine	+	+	+	+	+	+	+	+	+	+	+	>1024

Note: Different concentrations of extract and compounds evaluated using micro broth dilution method as recommended by NCCLS. All values are in µgmL^−1^. The (−) sign represents the absence of growth whereas the (+) sign represents the presence of growth.

## Data Availability

Not applicable.

## Sample Availability

All data are available and included in the manuscript.
